# Parental Reflective Functioning in Mothers and Fathers of Children With ADHD: Issues Regarding Assessment and Implications for Intervention

**DOI:** 10.3389/fpubh.2019.00263

**Published:** 2019-09-13

**Authors:** Claudia Mazzeschi, Livia Buratta, Alessandro Germani, Clarissa Cavallina, Roberta Ghignoni, Michele Margheriti, Chiara Pazzagli

**Affiliations:** ^1^Department of Philosophy, Social Sciences and Education, University of Perugia, Perugia, Italy; ^2^Istituto Agazzi “Futurabile”—Outpatient Center of the Developmental Age, Arezzo, Italy; ^3^Center for Clinical Neuropsychology of Developmental Age “Giorgio Sabbadini”, Perugia, Italy

**Keywords:** ADHD, co-parenting, parents' symptomatology, parental reflective functioning, assessment

## Abstract

Parental factors contribute to ADHD, partly in an etiological way and partly as moderators and mediators of child outcomes and treatment effects. An important aspect of parenting seems to be parental reflective functioning (PRF), defined as the parent's capacity to reflect upon his own and his child's internal mental experience. The studies on parenting factors linked to ADHD have not extensively investigated the role of PRF. Recent findings on interventions have begun to consider mentalization to promote empathy and emotion regulation in parents, but empirical studies assessing PRF are still scarce. The aim of this cross-sectional study was to compare specific familial and parental functioning characteristic between parents of children with attention deficit/hyperactivity disorder (ADHD) and parents of controls without ADHD. A clinical sample of 41 children with ADHD aged 8–11 years and their parents was compared with a matched, non-clinical sample of 40 children. Three aspects of parental functioning were investigated: parental symptomatology, parental alliances and PRF; children's differences in strength and difficulty profiles were also assessed. The results showed that families of children with ADHD had lower socioeconomic status, and both mothers and fathers of the same families reported higher scores for depression and lower PRF than did the control group; only mothers showed lower parental alliance. Logistic regression highlighted the fact that several of these familial and parental factors contributed to the increased risk of belonging to the clinical group, specifically both mothers' and fathers' depressive symptoms and lower PRF. These data represent new findings with potentially meaningful clinical implications for both assessment and intervention.

## Introduction

Attention deficit/hyperactivity disorder (ADHD) is one of the most prevalent neurodevelopmental disorders and constitutes a common cause for referral to psychological and psychiatric services. Although inattention and hyperactivity/impulsivity are core symptoms defining the disorder (1), it is characterized by a wide variability of symptoms and difficulties in functioning (2). Furthermore, it is known that impairments can be either alleviated or exacerbated by environmental factors (3). In fact, longitudinal studies have shown that ADHD expression is influenced by the complex interaction between genetic factors and environmental variables (4).

Because environmental factors can either alleviate or exacerbate the functional impairments caused by ADHD (3), studies have recently investigated the impact of specific parenting features on the expression and development of children's ADHD and on treatment effects (5–7).

In the last two decades, several studies have reported substantial differences in parental psychopathology and family functioning between families of children with ADHD and parents of no-ADHD controls (i.e., 6) (8). The effects are likely to be complex, and over time, both children and parents may be reinforced for maladaptive behaviors. On the one hand, the demanding, moody and uncooperative behavior described among children with ADHD can represent a parenting challenge and has an impact on parental behavior and adjustment. On the other hand, parenting characteristics and difficulties may exacerbate children's behavioral difficulties and the course of the disorder (9–12).

Studies on parental psychopathology characterizing families with ADHD have focused mainly on depressive disorders (13, 14). Recently, more attention has been paid to anxious-type symptoms (15, 16). Several studies have found that parents of children with ADHD report higher levels of depression and anxiety symptoms than do parents of children without ADHD (16, 17). Studies comparing mothers and fathers showed a greater presence of depression and anxiety symptoms in mothers than in fathers (18, 19). However, data are inconsistent because other studies did not find the same differences in psychopathology between parents of children with ADHD and parents of children in the control group, suggesting the need to further investigate this controversial issue (20). A recent meta-analysis showed that the research on these topics has largely focused on mothers, with fewer studies on fathers (21).

In regard to familial dimensions, Kaplan et al. (22) found that parents of children with ADHD reported more difficulties in family functioning. Families with ADHD are characterized by lower parental agreement or consistency regarding the management of their children (23) and higher levels of conflict in marital interactions [e.g., (23–25)]. Parents of children with ADHD are more discordant and conflictual than parents of controls, showing lower levels of parental alliance and more arguments about child-related and co-parenting issues (5, 26). However, few studies have focused on co-parenting alliances in families with ADHD.

In addition to studies on parents' psychopathological symptoms and parental alliances, research in the ADHD field has recently begun to empirically investigate the role of mentalization both in adults with ADHD and in parents of children with ADHD, as well as in some parenting interventions. Mentalization, operationalized as reflective functioning (RF), refers to the human capacity to understand behavior in light of underlying mental states and intentions. Impairments in this capacity have been implicated in a wide variety of disorders and behavioral problems (27).

In adults with ADHD, it has been at first assumed that RF impairments could be an important process underlying attentional, hyperactive/impulsive and emotional symptoms. Confirming this hypothesis, Perroud et al. (28) have recently found that adults with ADHD show impaired RF with respect to a healthy control group and that these impairments were intrinsically linked and correlated with attention and hyperactive/impulsive symptoms.

More recently, studies with parents of children with ADHD focused on parents' general capacity to reflect on mental states (i.e., RF) with more details on parental reflective functioning (PRF). It has been argued that the capacity to reflect on a specific relationship with a significant other could differ from more general mentalizing processes (29, 30). PRF is defined as parents' capacity to comprehend the developing mind of their child, reflect upon it and hold in mind the inner life of the child. As Sharp and Fonagy (29) noted, parents' capacity to engage their child in an accurate and appropriate way, is influenced by their own RF and the child's characteristics, especially their temperament. Previous studies have indicated that the parental capacity to give meaning to children's behavior shapes the parents' affective and behavioral reactions to the child (31). It has been hypothesized that the parents' capacity of children with ADHD to think about the mental processes underlying children's expressed emotions and behaviors, enhances their ability to contain the child's emotional and physical needs, leading to improvement in managing the child's distress (32, 33). In a sample of parents of children seeking ADHD treatment, Gershy and Gray (9) found that parents' capacity for mentalization serves as a buffer against parental hostile feelings, specifically among emotionally dysregulated parents. Parents' capacity to use mentalization while describing their child was assessed with mind-mindedness (34), using a single question interview in which parents were asked to describe their child. These findings highlight the potential role of parental mentalization as a protective mechanism in families of children with ADHD.

Recently, a more cost-effective and less time-consuming new measure to assess parents' capacity for mentalization has been validated. The Parental Reflective Functioning Questionnaire [PRFQ; (35)] is a brief and multidimensional assessment tool, quite different from mind—mindedness used in the previous study with parents of children with ADHD by Gershy and Gray (9). Studies that have assessed PRF using the PRFQ have shown that a parent's capacity to mentalize may be a critical factor in tolerating an infant's distress, enhancing more positive discipline strategies, and perceiving less parenting stress (33, 36–38). Hence, these studies showed that PRF may foster feelings of efficacy in dealing with distressing situations and interactions. As no research to date have assessed PRF with the PRFQ, and only one study has investigated mind mindedness in a sample of parents of children seeking ADHD treatment, studies exploring differences in PRF in parents of children with ADHD are needed.

Given the need to expand knowledge of the relationship between ADHD and parenting dimensions, specifically PRF, as a starting point, a cross-sectional and correlational study was carried-out. The aims of this study were as follows:

(1) To assess anxiety, symptoms of depression, and co-parenting alliances in both mothers and fathers, comparing parents of children with/without ADHD. Given that, until now, most extant research has focused mainly on mothers, to explore the possible role of fathers seems to be crucial;

(2) To investigate potential PRF impairments in parents of children with ADHD. Until now, no studies have compared parents' PRF in a clinical and control group. We hypothesized that the PRF scores of parents of children with ADHD would have been lower than those of control group;

(3) To further investigate the relation between the aforementioned parental and familial factors and the probability of increased risk of belonging to the clinical group. A higher probability of the presence of ADHD was expected to be related to higher parental symptomatology, lower parental alliances, and lower levels of PRF.

## Methods

### Participants and Procedure

In order to estimate the sample size, power analysis was carried-out using G^*^Power 3.1 (39). Three factors were considered, both for logistic regression and MANOVA. With regard to logistic regression, odds ratio = 2 (p H1 = 0.25, p H0 = 0.15), α = 0.05, and power = 0.90 were selected. Power analysis indicated that there was a 90% chance of correctly rejecting the null hypothesis that predictor variable was not associated with outcome variable, with a sample of 168 participants. As to MANOVA, a significant level of 0.05, a small effect size for a conservative approach (*f* ^2^ = 0.10), and a power of 0.90 were considered. Power analysis indicated a total sample size of 100 participants. Thus, one hundred and seventy-eight parents of children aged 8–11 were recruited.

Eight children belong to the control group were excluded for data analysis due to presence of difficulties referred by their parents or because they were under psychologically treatment. Therefore, the sample included 162 parents of 81 children aged 8 and 11 years (69.1% males); see Table 1.

**Table 1:** Descriptive statistics in terms of means and standard deviation and frequencies for sample description.

	**Clinical group**			**Control group**					
	**N**	**Mn**	**SD**	**N**	**Mn**	**SD**	***F***	**Δ (95% CI)**	***$\eta_{p}^{2}$***
**Children**									
Male	34			22					
Female	7			18					
Age	41	9.37	1.68	40	9.55	0.57	0.426	0.184 (−0.378/0.746)	0.005
SDQ hyperactivity-inattention	41	4.39	1.37	40	2.85	1.39	20.31	−1.54 (−2.21/−0.860)^*^	0.204
**Family**									
Mothers age	41	40.29	2.72	40	42.03	6.48	2.48	1.73 (0.119/−0.457)	0.030
Fathers age	41	47.21	4.50	40	48.80	3.62	3.01	1.59 (−0.234/3.42)	0.038
SES	41	36.78	7.69	40	41.05	7.81	6.14	4.27 (0.841/7.69)^*^	0.070

*ANOVA shows differences between the clinical (41) and control groups (40)*.

* *p < 0.05 significant difference*.

$\eta_{p}^{2}$*: ≥0.0099 small effect size; ≥0.0588 medium effect size; ≥0.1379 large effect size*.

*SES, socioeconomic status; SDQ, strength and difficulties questionnaire*.

The clinical group consisted of 41 children (mean age = 9.37 years; SD = 1.68), including 34 males and 7 females and their parents enrolled from two clinical centers in central Italy specialized in the assessment and treatment of neurodevelopmental disorders. The clinical group was selected by means of the director of the centers from their clinical populations, on the basis of the following inclusion criteria: (a) children having ADHD at their first diagnosis, according to DSM-5 criteria; (b) children having an IQ > 70; and (c) parents having good knowledge and fluency of the Italian language. All of the cases selected participated in the study and completed the measures at the center during a visit within the assessment phase. For the clinical group, the mean age of mothers was 40.29 years (SD = 2.72), and the mean age of fathers was 47.21 years (SD = 4.74).

The control group consisted of 40 children (22 males and 18 females) matched with the clinical group on age (mean = 9.55; SD = 0.56; *F* = 0.426, *p* = 0.516), as well as their parents. They were recruited through two public schools in the same region. Parents were asked to participate in the study by the teachers and were enrolled through convenience sampling in three different classes. The response rate among cases was 96%. The mean age of mothers was 42.03 years (SD = 6.48), and the mean age of fathers was 48.80 years (SD = 3.48). All the parents participating in the study completed a sociodemographic questionnaire according to Hollingshead's Four Factor Index of Social Status (40), a general form on the child regarding the presence of any illness or disability (either physical or mental) or any possible problems at school, and some questionnaires (see Measures paragraph).

Families had a middle level of socioeconomic status (SES) in both the clinical group (mean = 36.78; SD = 7.68) and the control group (mean = 41.05; SD = 7.81). ANOVA showed significant differences between the two groups (*F* = 4.72; *p* = 0.033; $\eta_{p}^{2}$ = 0.056).

Children filled in the Italian version of the Strengths and Difficulties Questionnaire [SDQ; (41)] as behavioral screening to control for differences in strength and difficulty profiles. Differences were found for the hyperactivity-inattention subscale between the clinical and control group (*F* = 20.31; *p* < 0.001; $\eta_{p}^{2}$ = 0.20). The control group showed scores within the normal range. All the participants were Caucasian. Data were collected after the parents' sign of the informed consent, according to the Ethical Principles of Psychologists and Code of Conduct of the American Psychological Association (42). Approval by the Ethical Committee for Psychological Research at the Department of Philosophy and Social Sciences and Education—University of Perugia was obtained, in line with the Italian Association of Psychology (AIP) Code of Conduct.

## Measures

### Parents' Measures

*State and Trait Anxiety Inventory—Y* [STAI-Y; (43)]: it is a self-report consisting of 40 items that measure two kinds of anxiety using a 4-point Likert scale ranging from 1 (not at all) to 4 (very much so). Twenty items assess state anxiety (or anxiety about a specific moment or event), and 20 items assess trait anxiety (or anxiety as a personal characteristic). The STAI has good internal consistency, test-retest reliability for the STAI Trait scale, sensitivity to the detection of stress for the STAI State scale, and convergent and discriminant validity (43). The Italian version of the STAI—Y (44) was used, showing good internal consistency and adequate test-retest reliability.

*Center for Epidemiologic Studies Depression Scale* [CES-D; (45)]: it is a brief self-report consisting of 20 items on symptoms of depression developed to measure depression severity in the general population. According to the measure, parents were asked to respond using a 4-point Likert scale, ranging from 0 (rarely) to 3 (all of the time). The Italian version of the CES-D was used (46). The Italian version of the CES-D exhibits adequate internal consistency.

*Parental Alliance Measure* [PAM; (47)]: a 20-item self-report questionnaire that assesses the parenting aspects of a couple's relationship in terms of co-parenting alliance (the communication, levels of cooperation and mutual respect they exhibit with regard to their children's care) using a 5-point Likert scale that ranges from 1 (strongly disagree) to 5 (strongly agree). The Italian version was used (48). Its Cronbach's alphas show good internal consistency.

*Parental Reflective Functioning Questionnaire* [PRFQ; (35)]: it is a self-report measure consisting of 18 items, divided into three subscales that assess PRF. The *pre-mentalizing* (PM) subscale assesses parental difficulty in understanding and interpreting the child's mental experience; the *certainty about mental states* (CMS) subscale evaluates the parents' inability to recognize the children's mental state as readily apparent; and the *interest and curiosity* subscale (IC) assesses the parents' ability to think about the child's internal experiences and to take the child's perspective. A 7-point Likert scale from 1 (strongly disagree) to 7 (strongly agree) is used to score each item. The Italian version of the PRFQ was used, and its Cronbach's alpha exhibited good to acceptable internal consistency in both mothers and fathers separately (30).

## Data Analysis

To investigate differences between parents of children with and without ADHD, multivariate analysis of variance (MANOVA) was used on parents' symptomatology (depression and anxiety) and familial and parental measures for mothers and fathers separately, with SES as covariates. Effect size was measured using partial eta-squared, in which small, medium, and large effects were 0.0099, 0.0588, and 0.1379, respectively [(49), p. 283].

In order to understand how much, parental and familial functioning levels increased the risk of belonging to the clinical group, logistic regression analyses were performed on the whole sample. All analyses were performed using SPSS, release 18 (50).

## Results

MANOVA showed a significant multivariate main effect of group (clinical vs. control group) on parents' depressive symptomatology, parental alliance, and PRF, both for mothers (Wilks' λ = 0.423, *F*_(1, 80)_ = 13.84, *p* < 0.001, $\eta_{p}^{2}$ = 0.577) and fathers (Wilks' λ = 0.501, *F*_(1, 80)_ = 9.658, *p* < 0.001, $\eta_{p}^{2}$ = 0.499). Mean, standard deviation and the univariate main effect are shown in Tables 2, 3. Both mothers and fathers of the clinical group reported higher level of depression than those of the control group, with medium effect size. There were no differences for anxiety symptoms. Regarding familial factors, only the mothers of children with ADHD reported lower levels of the parental alliance than those of children without ADHD, with small effect size. Concerning PRF, mothers of clinical group referred higher PM and CSM, as well as higher IC levels than those of control group, with medium-large effect sizes. Fathers of clinical group referred higher CSM and lower IC levels than those of control group, with medium and large effect sizes, respectively. Whereas, they reported similar PM levels.

**Table 2:** Multivariate analysis of variance (MANOVA) for group (clinical group = 41; control group = 40) with means and standard deviations for STAI, CES, and PAM.

	**Clinical group**		**Control group**				
	**Mn**	**Sd**	**Mn**	**Sd**	***F_**(1, 80)**_***	**Δ (95% CI)**	***$\eta_{p}^{2}$***
**Mothers**							
STAI trait	42.32	6.42	42.00	6.95	0.281	−0.82 (−3.91/2.26)	0.004
STAI state	37.60	8.46	37.27	7.969	0.354	−1.10 (−4.81/2.60)	0.005
CES-D	13.37	11.95	6.27	8.10	10.00	−7.52 (−12.25/−2.78)*	0.115
PAM	74.40	17.93	83.60	13.02	5.14	8.24 (1.00/15.48)*	0.063
**Fathers**							
STAI trait	39.38	7.11	37.22	5.95	1.69	−2.03 (−5.13/1.08)	0.001
STAI state	34.86	7.12	34.47	7.94	0.044	1.80 (−3.97/3.21)	0.022
CES-D	12.19	11.72	4.82	6.40	11.57	−7.57 (−12.01/−3.14)*	0.135
PAM	85.27	12.60	84.92	10.47	0.163	−1.10 (−6.55/4.34)	0.002

F and associational estimates were reported. *p < 0.05 significant difference.

$\eta_{p}^{2}$*: ≥0.0099 small effect size; ≥0.0588 medium effect size; ≥0.1379 large effect size*.

*STAI, state and trait anxiety inventory; CES-D, center for epidemiologic studies depression scale; PAM, parental alliance measure*.

**Table 3:** Multivariate analysis of variance (MANOVA) for group (clinical group = 41; control group = 40) with means and standard deviations for the PRFQ scales.

	**Clinical group**		**Control group**				
	**Mn**	**Sd**	**Mn**	**Sd**	***F_**(1, 80)**_***	**Δ (95% CI)**	***$\eta_{p}^{2}$***
**Mothers**							
**PRFQ**							
PM	2.55	1.42	1.97	0.63	4.53	−0.55 (−1.06/−0.03)^*^	0.058
CMS	3.84	1.39	2.96	0.39	13.23	−0.87 (−0.1.34/−0.39)^*^	0.147
IC	4.92	1.51	6.39	0.32	30.24	1.39 (0.884/1.88)^*^	0.282
**Fathers**							
**PRFQ**							
PM	2.36	1.08	1.96	0.48	3.73	−0.38 (−0.78/0.01)	0.048
CMS	3.38	1.18	2.88	0.36	5.81	−0.49 (−0.901/−0.08)^*^	0.073
IC	5.08	1.32	6.14	0.48	19.31	1.03 (0.56/1.49)^*^	0.207

*F and associational estimates were reported*.

* *p < 0.05 significant difference*.

$\eta_{p}^{2}$*: ≥0.0099 small effect size; ≥0.0588 medium effect size; ≥0.1379 large effect size*.

*PRFQ, parental reflective functioning questionnaire; PM, pre-mentalizing; CMS, certainty about mental states; IC, interest and curiosity*.

Table 4 shows a logistical regression analysis in which familial and parental factors for both mothers and fathers were entered as predictors of ADHD. The results highlighted that several familial and parental factors contribute significantly to the increased risk of belonging to the clinical group. In particular, in the mothers group, an OR of 1.07 (95% CI = 1.02–1.13) indicated a significant increase (β = 0.07, *p* < 0.01) in the odds of being in the clinical group for each unit increment of CES. The same significant effect was found in the fathers group (OR = 1.10; 95% CI = 1.02–1.17; β = 0.09, *p* < 0.01). Regarding the PRFQ subscales in the mothers group, the OR of 9.75 (95% CI = 2.59–36.71) showed a significant increase (β = 2.28, *p* < 0.001) in the odds of being in the clinical group for each unit increment of CMS. The same significant increase was observed in the fathers group (OR = 5.10; 95% CI = 1.79–14.50; β = 1.63, *p* < 0.001); however, the large CI reduced the reliability as a factor risk of both mothers' and fathers' CMS. With respect to the co-parenting alliance in the mothers group, the OR of 0.96 (95% CI = 0.933–0.992) indicated a significant decrease (β = −0.04, *p* < 0.05*)* in the odds of being in the clinical group for each unit increment of PAM. No significant effect was observed for fathers' perceptions of co-parenting alliances. Furthermore, in the mothers group, the OR of 0.06 (95% CI = 0.012–0.295) showed a significant decrease (β = −2.83 *p* < 0.001) in the odds of being in the clinical group for each unit increment of the IC subscale of the PRFQ. The same results were found for fathers (OR = 0.151; 95% CI = 0.056–0.411; β = −1.89, *p* < 0.001). Neither anxious symptomatology nor the PM subscale of the PRFQ in both parents contributed to ADHD.

**Table 4:** Logistic regression analysis of socioeconomic status and parental and familial measures on the presence of ADHD of the whole sample.

	**β**	**χ^2^**	**Wald statistics**	**OR**	**95% CI**
**Mothers**					
STAI		0.28			
STAI trait	0.005		0.013	1.00	0.927–1.08
STAI state	0.000		0.000	1.00	0.917–1.10
CES-D	0.07	8.94^**^	7.29^**^	1.07	1.02–1.13
PAM	−0.04	6.79^**^	5.93^*^	0.96	0.933–0.992
PRFQ		62.29^***^			
PM	−0.561		0.700	0.57	0.153–2.12
CMS	2.28		11.34^***^	9.75	2.59–36.71
IC	−2.83		11.89^***^	0.06	0.012–0.295
**Fathers**					
STAI		2.60			
STAI trait	0.082		2.44	1.09	0.979–1.20
STAI state	−0.48		1.15	0.95	0.872–1.04
CES-D	0.093	10.86^***^	7.12^**^	1.10	1.02–1.17
PAM	0.003	0.018	0.018	1.00	0.964–1.04
PRFQ		37.611^***^			
PM	−0.47		0.691	0.625	0.206–1.89
CMS	1.63		9.33^**^	5.10	1.79–14.50
IC	−1.89		13.68^***^	0.151	0.056–0.411

*STAI, state and train anxiety inventory; CES-D, center for epidemiologic studies depression scale; PAM, parental alliance measure; PRFQ, parental reflective functioning questionnaire; PM, pre-mentalizing; CMS, certainty about mental states; IC, interest and curiosity*.

* *Effect was statistically significant at p < 0.05*.

** *Effect was statistically significant at p < 0.01*.

*** *Effect was statistically significant at p < 0.001*.

## Discussion

The first aim of this study was to examine possible differences in symptoms of anxiety and depression and in co-parenting alliances between parents of children with ADHD and parents of control children. Results showed significant differences in parents' depressive symptomatology with medium effect size. Both mothers and fathers of clinical group reporting higher levels than those of control group. Until now, literature on parental psychological aspects seems to be inconsistent: numerous studies have reported more affective disorders in the relatives of children with ADHD than in the families of control children, specifically for depressive symptoms (51). However, Johnston and Mash (5) highlighted that the association between parental affective disorder and child ADHD is not as strong. Some studies have not found differences in either the mothers or fathers of children with ADHD or the parents of control children (20, 52). Furthermore, only a few studies have focused on paternal psychopathology (53, 54). The present study, according to previous data, showed higher levels of internalizing disorder, specifically depressive symptoms, in both mothers and fathers of children with ADHD. No differences emerged with regard to anxious symptoms in mothers and fathers. These results seem to be in line with evidence of a greater risk of behavioral problems, including ADHD, in children of mothers with depression (20, 55, 56). These findings suggest the importance of considering fathers' depressive symptoms in further studies, as well as in clinical setting, because it seems that it is an important factor in families of children with ADHD.

Referring family functioning, not many studies have focused specifically on co-parenting alliances. Research has documented the relation between inter-parental conflict and child behavioral problems in families with more frequent arguments regarding child-related issues (26, 57). Couples of relatives of youth with ADHD are more discordant over collaborative parenting issues than are couples who are parents of children without ADHD (5). These co-parenting difficulties could be related to significant difficulties in the management of the child (58). The present result on co-parenting alliances as perceived by parents, showed lower level of parental alliance in mothers of children with ADHD than in mothers of children in the control group. No differences between the fathers of the two groups emerged. These data could be an expression of the specific maternal parenting role and of the greater amount of time mothers often spend interacting with their children (23). Further studies may be needed to analyse this discrepancy between mothers and fathers regarding perceptions of co-parenting alliances, as the parental alliance constitutes an important factor for success in family interventions (59).

Although recent research indicates the importance of mentalization in the ADHD field, both in empirical studies and in parenting interventions, only one study to date has investigated this issue in families of children with ADHD, showing that parental mentalization could act as a buffer against parental hostility (9). Recent research demonstrating the relationship between PRF and parents' capacity to regulate their own emotions in the caregiving context indicates the importance of the meaning parents apply to children's behaviors to determine the emotional/physiological level of arousal the parent experiences in reaction to them (9). Drawing upon these findings and upon studies showing more parenting and familial difficulties among parents of children with ADHD, the second aim of this study was to explore potential PRF impairments in parents of children with ADHD and to compare these impairments with those of a control group of parents. As expected, significant differences emerged in PRF between the two groups. Specifically, both mothers and fathers of children with ADHD, compared to the control group, showed: (a) a significantly higher non-mentalizing stance, showing more difficulties in entering the subjective world of the child; (b) a significantly higher level of certainty in mental states, showing a greater tendency toward unjustified assumptions about their child's states of mind; and (c) a significantly lower level of genuine interest and curiosity in their child's mind. Taken together, this specific combination of PRFQ scales suggested that parents of children with ADHD, in comparison to parents of children in the control group, showed greater difficulties in recognizing the opaqueness of children's mental states and in understanding that they have a limited ability to truly know what is in their child's mind. These difficulties in tolerating the uncertainty that occurs from not knowing why the child is behaving in a certain way seemed to be associated with lower genuine interest and curiosity in the child's mental states. Furthermore, parents' difficulties in understanding why their children act or feel differently from their expectations also emerged from the more high-level non-mentalizing stance, which expressed parents' tendency to make maladaptive and malevolent attributions about the child and, broadly, to repudiate or defend against mentalizing.

Overall, parents of children with ADHD showed more difficulties in PRF capacities than parents of children without ADHD. As PRF is considered a key feature of adaptive parenting and of fostering feelings of efficacy in dealing with distressing interactions (60), these data seemed to be in line with the differences emerged in this study between the two groups in depression symptoms and parenting alliances. The presence of both parents' depression symptomatology, maternal perceptions of a low co-parenting alliance, and low PRF also played a significant role as risk factors of belonging to the clinical group, as investigated in the third aim of this study.

Difficulties in parents' capacity to give meaning to children's behavior shape the parents' affective and behavioral reactions to the child, making parents feel more helpless, fatigued and unsupported. As Sharp and Fonagy (29) noted, the parental capacity to engage in accurate and appropriate mentalizing (PRF) is considered to be also influenced by child characteristics, and mutual affect regulation and attunement are assumed to characterize dyadic interactions from birth onwards. The effects are likely to be complex, and over time, both children and parents may be reinforced for maladaptive behaviors. As outlined by Nijssens et al. (38), the lack of feelings of control and efficacy that may be experienced by parents with poorer PRF could entail an increasing belief that interactions spiral out of control, an issue frequently described in families with children with ADHD. Furthermore, the present finding on PRF substantiated the increasing attention paid to the role of mentalization both in empirical studies and in parenting interventions in the ADHD field, highlighting the potential role of enhanced parental capacity to think about the mental processes underlying children's expressed emotions and behaviors in families of children with ADHD.

Taken together, these findings on PRF add to the body of knowledge about the role of sensitive, supportive parenting in the developmental pathways through which child and family characteristics transact to exert their influences over time (5).

Furthermore, as the majority of the research on parental features as a risk or protective factor for children's developmental outcomes has investigated mothers, the present findings highlight the need for further study of the father-child relationship, which has in the past been somewhat neglected in comparison to the mother-child relationship (61).

Several limitations must be addressed in the present study. The main limitation is that it is based on self-report questionnaires. Further replication of the findings from this study is therefore needed with interviews and observer-based measures. Furthermore, results cannot support causal relationships among variables and no-ADHD/ADHD given that the data were cross-sectional in nature. Finally, given the small size of the group because the disorder is very rare, the results of this study must be interpreted with caution, and more research in larger groups is needed, even though sample size was adequate according to power analysis.

Overall, the results confirmed previous studies on substantial differences between families of children with ADHD and parents of no-ADHD controls in parental psychopathology and parental functioning (7). This study adds to previous research on PRF, showing more difficulties among both parents in understanding the underlying reasons for the child's behavior, with respect to the control group. The difficulties that emerged in PRF capacities may bear clinical significance in suggesting early interventions targeting PRF. With respect to parenting interventions, studies have shown that improvement in mothers' insightfulness was associated with a decrease in children's behavior problems (62). Moreover, the most recent family-based intervention approaches for ADHD have begun to consider mentalization to promote empathy and emotion regulation in parents and their children, but studies are still scarce (63). Considering the difficulties that parents of children with ADHD face and the effect of parenting dysfunction on children, several interventions for parents of children with ADHD have been developed, mostly of a psycho-educational style and cognitive behavioral therapy orientation, focusing largely on guidance and skill training (64–67). In contrast, the present findings suggest as a focus of treatment the parents' capacity to envision their child as being motivated by internal mental states and to be able to reflect on their own internal mental experiences and how they are shaped and changed by interactions with the child (35).

## Data Availability

The datasets for this manuscript are not publicly available because for privacy and ethics reasons. Requests to access the datasets should be directed to CM.

## Ethics Statement

Data were collected after parents had signed the informed consent form to participate to the study, according to the Ethical Principles of Psychologists and Code of Conduct of the American Psychological Association (42). Approval by the Ethical Committee for Psychological Research at the Department of Philosophy and Human Sciences of Perugia University was obtained, in line with Italian Association of Psychology (AIP) Code of Conduct.

## Author Contributions

CM: substantial contribution to the conception of the work, to the research design, to data collection and interpretation, drafting the work, and final approval of the version. CM will be accountable for all aspects of the paper. LB: substantial contribution to data analysis, drafting the paper, and approval of the version to be published. AG: contribution to data analysis and agreement to be accountable for all aspects of the paper. CC, RG, and MM: contribution to data collection and agreement to be accountable for all aspects of the paper. CP: substantial contribution to data collection and interpretation, drafting the work, approving the final version, and agreement to be accountable for all aspects of the paper.

### Conflict of Interest Statement

The authors declare that the research was conducted in the absence of any commercial or financial relationships that could be construed as a potential conflict of interest.
